# Patients with more complex ankle fractures are associated with poorer patient-reported outcome: an observational study of 11,733 patients from the Swedish Fracture Register

**DOI:** 10.2340/17453674.2024.40607

**Published:** 2024-05-07

**Authors:** Caroline STIGEVALL, Michael MÖLLER, David WENNERGREN, Olof WOLF, Jan EKELUND, Carl BERGDAHL

**Affiliations:** 1 Institute of Clinical Sciences, Sahlgrenska Academy, University of Gothenburg, Gothenburg; 2 Department of Orthopedics, Sahlgrenska University Hospital, Gothenburg/Mölndal; 3 Department of Surgical Sciences, Orthopedics, Uppsala University, Uppsala; 4 Center of Registers Västra Götaland, Gothenburg, Sweden

## Abstract

**Background and purpose:**

Patient-reported outcome measures (PROMs) following ankle fractures, including all fracture types, have not been reported. It is therefore unclear whether fracture morphology correlates with outcome. We aimed to analyze PROMs in patients with an ankle fracture in relation to the Arbeitsgemeinschaft für Osteosynthesefragen/Orthopaedic Trauma Association (AO/OTA) fracture classification using population-based register data from the Swedish Fracture Register (SFR).

**Methods:**

All patients aged ≥ 18 years with an ankle fracture (AO/OTA 44A1–C3) registered in the SFR between 2012 and 2019 were retrieved from the register. Patients with completed PROM questionnaires (Short Musculoskeletal Function Assessment and EuroQol-Visual Analogue Scale) on both day 0 (pre-trauma) and 1-year post-trauma were included. The difference in PROMs between day 0 and 1 year was calculated for each patient (delta value) and mean delta values were calculated at group level, based on the AO/OTA fracture classification.

**Results:**

11,733 patients with 11,751 fractures with complete PROMs were included. According to the AO/OTA classification, 21% were A fractures, 67% were B fractures and 12% were C fractures. All groups of patients, regardless of fracture class (A1–C3), displayed an impairment in PROMs after 1 year compared with day 0. Type C fractures displayed a larger impairment in PROMs at group level than type B, which in turn had a greater impairment than type A. The same pattern was seen in groups 3, 2, and 1 for A and B fractures.

**Conclusion:**

We found that the AO/OTA classification is prognostic, where more complex fractures were associated with poorer PROMs.

The ankle is the third most common fracture location in the adult population, and the incidence has been shown to increase during the last few decades [1-4]. There is great diversity among ankle fractures, with a varying degree of severity, which most likely results in different outcomes. In clinical practice, fractures are therefore grouped into different classifications that serve as an essential basis for fracture management. A classification system of ankle fractures needs to consider not only fracture morphology but also the ligamentous lesions.

Previous studies of outcome following ankle fractures have focused on either one type of treatment or a specific type of ankle fracture [5-7] but there are no studies of patient-reported outcome of all types, regardless of treatment. To give future patients a realistic expectation on outcome after their ankle fracture, it is of great value to provide patients and healthcare providers with data based on patient-reported outcome. Therefore, it would be of value to report the outcome following ankle fractures, in relation to fracture classification. The hypothesis is that a more severe fracture morphology correlates with worse outcome.

The aim of this study was to analyze patient reported-outcome measures (PROMs) in patients with ankle fractures in relation to the AO/OTA (Arbeitsgemeinschaft für Osteosynthesefragen/Orthopaedic Trauma Association) fracture classification, using population-based data from the Swedish Fracture Register (SFR).

## Methods

This study is an observational register study of prospectively collected data retrieved from the SFR. It is reported according to STROBE guidelines.

The Swedish Fracture Register (SFR) is a unique national quality register that prospectively collects data on all fractures, regardless of treatment. The SFR started at Sahlgrenska University Hospital in 2011 and was gradually expanded to reach full coverage of the country’s orthopedic departments in 2021. Details for all patients aged ≥ 18 years, with an ankle fracture (i.e., AO/OTA classification 44A1–C3), regardless of treatment, sustained between 2012 and 2019, were retrieved from the SFR. Data on demography (patient age and sex), injury (high- or low-energy trauma, open or closed, and date), fracture (fracture type and group according to the AO/OTA classification, treatment modality, and treatment date), and PROMs were retrieved from the SFR. 3 different PROMs are used in the SFR of which 2 are used in this study: the Short Musculoskeletal Function Assessment (SMFA) and EuroQol-Visual Analogue Scale (EQ-VAS). Baseline data are collected using the recall technique (day 0) and patients who respond receive a new questionnaire 1 year after injury. The implementation, design, validation, and registration process of the SFR have previously been described in detail [8-10]. Patients with PROM data for the EQ-VAS or at least one of the sub-indices in the SMFA, both day 0 and 1 year, were included in the study. Patients with concomitant fractures on the same trauma occasion or another ankle fracture within a year of the primary fracture were excluded.

The SMFA is a validated self-reported health questionnaire with high reliability and good validity used in patients with musculoskeletal disorders or injuries [11-13]. The questionnaire consists of 2 main indices. The Dysfunction index assesses function, and the Bother index assesses how bothered the patient is during common daily activities. The Dysfunction index is further sectioned into the categories of daily activities, function of the arm and hand, mobility, and emotional status. The index scores range from 0 to 100, where lower scores indicate better function [11]. In this study, Dysfunction and Bother indices of the SMFA are analyzed and also the sub-category Mobility from the Dysfunction index, as it is the sub-category that is theoretically most affected following an ankle fracture.

The EQ-VAS is used as a quantitative measurement of health that reflect the patient’s own assessment of their health with a single value. The patient is asked to estimate their overall health status on a scale ranging from 0 to 100, where 0 represents the worst imaginable health and 100 the best imaginable health [14].

### Statistics

The Dysfunction and Bother indices of the SMFA, the sub-category Mobility from the Dysfunction index, and EQ-VAS were statistically analyzed with the aim of estimating effects of fracture classification, age groups, sex, mechanism of injury, and open or closed injury on the change (delta value) in PROM from day 0 to 1 year after injury.

**Figure 1:**
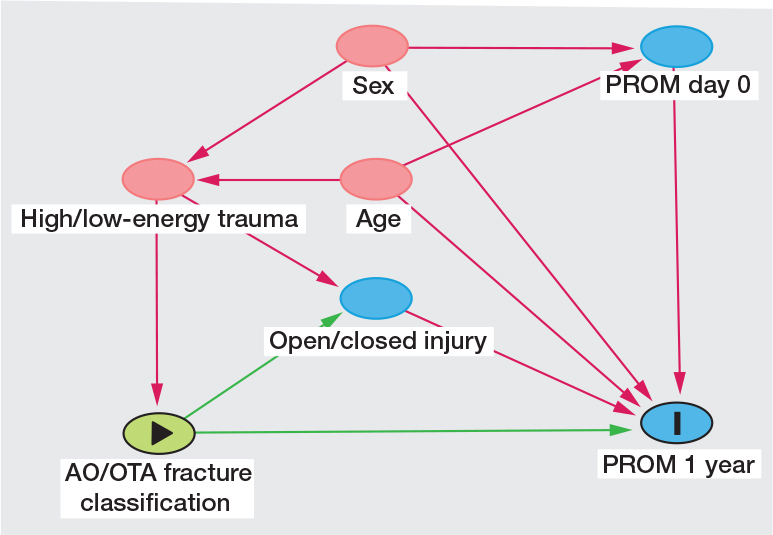
Directed acyclic graph (DAG). Green arrow is causal path and pink arrow biasing path

To estimate the effect of fracture classification, the change in each PROM was analyzed using an analysis of covariance (ANCOVA) with fixed effects for the factors sex, age groups, mechanism of injury, open or closed injury, and fracture classification (type, type and group in 2 separate analyses), and with the day 0 value for the respective PROM as a continuous covariate. This model was also used to estimate the effect of mechanism of injury and the effect of open or closed injury. The assumed relationship between explanatory variables and the dependent variable is described in the directed acyclic graph (DAG) (Figure 1).

To estimate the effects of sex within each age group, an ANCOVA with the same factors and covariate as previously described was extended with the interaction between age groups and sex. The effect of open or closed injury for each mechanism of injury was further estimated using the same model as previously described, extended with the interaction between mechanism of injury and open or closed injury.

The results are presented as estimated marginal means with 95% confidence interval (CI) for each individual factor level and estimated differences between factor levels are presented with CIs. For estimation of the marginal means, covariates are held at their overall mean value. Because of the sample size, estimates are considered to be approximately normally distributed and confidence intervals have an approximate confidence level of 95%. Descriptive statistics are presented as mean and standard deviation (SD) for continuous variables and number and percentage for categorical variables. SPSS statistics, version 28.0 (IBM Corp, Armonk, NY, USA) was used for the statistical analysis.

### Ethics, data sharing, funding, and disclosures

This study was conducted in accordance with the Declaration of Helsinki and approved by the Swedish Ethical Review Board Authority (DNR 2021-00513).

The data from the SFR in this study was obtained after approval from the Swedish Ethical Review Board Authority with the assurance of the confidentiality of included patients. The data is therefore not publicly available in accordance with the law on Public Access and Security [15]. There are ways to share data according to Swedish law and interested persons can contact Gothenburg University and the corresponding author for more information. The participants in this study have no potential conflicts of interest or funding to declare. Complete disclosure of interest forms according to ICMJE are available on the article page, doi: 10.2340/17453674.2024.40607

## Results

**Figure 2:**
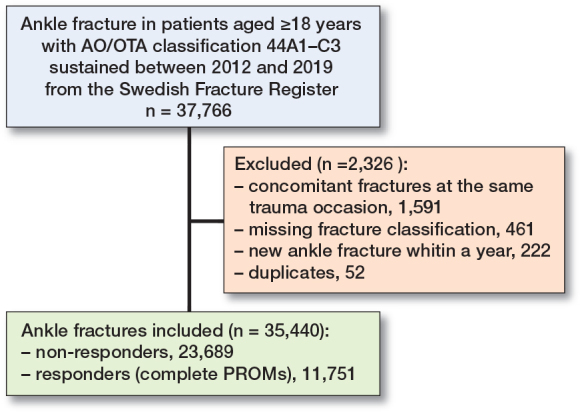
Flowchart of ankle fractures included in the study. PROM = patient-reported outcome measures.

During the study period 2012–2019, 37,766 ankle fractures were registered in the SFR in patients aged ≥ 18 years (Figure 2). 461 fractures had a missing fracture classification and 52 were duplicates and were subsequently excluded. There were 1,591 patients with concomitant fractures and 222 fractures occurred in patients within a year of a previous ankle fracture and these were also excluded. Of the remaining 35,440 ankle fractures, 23,689 were excluded due to missing PROM data on either day 0 or 1 year after fracture (i.e., non-responders). 11,751 fractures in 11,733 patients were therefore available and included in the study.

The patients (i.e., responders to PROM questionnaires) had a mean age of 58 (SD 16) years and the majority were women (65%) (Table 1). Low-energy trauma was the dominant mechanism of injury (87%) and slightly more patients were treated surgically (54%) than non-surgically. The highest number of ankle fractures was seen in the age group 61–70 years, which accounted for 27% of all ankle fractures. The B-type fracture was the most common fracture (67%) (Table 2).

**Table 1:** Baseline characteristics of responders (i.e., study cohort) and non-responders to patient-reported outcome measures on ankle fractures. Values are percentages unless otherwise specified

Factor	Responders	Non-responders
Ankle fractures, n	11,751	23,689
Male sex	35	42
Mean age (SD)	58 (16)	54 (20)
High-energy	3.8	3.9
Low-energy	87	86
Trauma mechanism unknown	9.0	11
Open injury	1.9	1.7
Surgical treatment	54	46
AO/OTA classification		
A	21	25
B	67	63
C	12	12
Age groups, n (%)		
18–30	986 (8.4)	3,736 (16)
31–40	776 (6.6)	2,463 (10)
41–50	1,456 (12)	3,544 (15)
51–60	2,612 (22)	4,441 (19)
61–70	3,133 (27)	4,092 (17)
71–80	2,085 (18)	3,058 (13)
≥ 81	703 (5.9)	2,355 (10)

**Table 2:** Distribution of the 11,751 ankle fractures included in the study according to the AO/OTA classification, type, and group. Values are count and (%)

Fracture type	Fracture group			
	1	2	3	Total
A	1,708 (15)	588 (5.0)	161 (1.4)	2,457 (21)
Surgery^a^	(5)	(55)	(65)	(17)
B	4,273 (36)	1,689 (14)	1,887 (16)	7,849 (67)
Surgery^a^	(32)	(89)	(96)	(60)
C	632 (5.4)	383 (3.3)	430 (3.7)	1,445 (12)
Surgery^a^	(77)	(96)	(85)	(85)
Total	6,613 (56)	2,660 (23)	2,478 (21)	
Surgery^a^	(29)	(82)	(92)	

a Proportion (%) of patients with surgical treatment within group.

There were small discrepancies in demography between the 11,751 responders and the 23,689 non-responders (Table 1). There was a larger proportion of women (65% vs. 58%) and a higher mean age (58 [SD 16)] vs. 54 [SD 20] years) in the responder cohort. The responder cohort were more often surgically treated (54% vs 46%), while there were only marginal discrepancies in the distribution of ankle fractures according to fracture classification (A1–C3) between the 2 groups.

### PROMs in relation to age groups and sex

All age groups demonstrated impaired mean delta values for all PROMs 1 year after injury, with positive mean delta values for the SMFA and negative for the EQ-VAS (Table 3). Overall, there was a tendency towards greater impairment in patients ≥ 81 years of age for practically all PROMs compared with patients of a younger age. For the 2 SMFA indices, the largest impairment in the Bother index was seen in the age groups 18–30 and ≥ 81 years for both men and women. The largest impairment in the Dysfunction index was seen in the oldest age group (≥ 81) where there was a 14.7 (CI 12.9–16.5) points impairment in women and 11.5 (CI 9.3–13.7) points in men. The sub-category Mobility showed the largest impairment in both sexes in patients aged ≥ 81 years, with an impairment of 17.5 (CI 15.2–19.8) points among women and 14.4 (CI 11.6–17.1) points in men. The EQ-VAS value showed the largest impairment of mean delta values in the age groups ≥ 81 years in both sexes.

**Table 3:** Mean delta values (∆) and (CI) of PROMs with ANCOVA^a^, 1 year after ankle fracture compared with day 0 in relation to age groups and sex

Factor Age	Sex	Bother (CI)	∆SMFA-index Dysfunction (CI)	Mobility (CI)	∆EQ-VAS (CI)
18–30	M	10.3 (8.0–12.5)	7.4 (5.6–9.2)	10.2 (7.9–12.4)	–8.7 (–12.1 to –5.4)
	F	11.8 (9.5–14.0)	8.6 (6.8–10.4)	12.4 (10.2–14.6)	–11.6 (–15.0 to –8.3)
31–40	M	9.2 (6.8–11.7)	6.7 (4.8–8.6)	10.0 (7.6–12.4)	–9.4 (–13.0 to –5.9)
	F	10.9 (8.5–13.2)	8.1 (6.2–9.9)	12.3 (10.0–14.7)	–11.3 (–14.8 to –7.8)
41–50	M	9.8 (7.6–12.0)	7.9 (6.2–9.7)	11.3 (9.2–13.5)	–9.5 (–12.8 to –6.2)
	F	10.9 (8.8–13.0)	8.7 (7.0–10.4)	13.4 (11.3–15.5)	–10.5 (–13.7 to –7.3)
51–60	M	9.9 (7.8–12.0)	7.4 (5.7–9.0)	11.0 (8.9–13.0)	–8.2 (–11.4 to –5.0)
	F	9.8 (7.9–11.8)	8.1 (6.5–9.6)	12.6 (10.7–14.6)	–9.8 (–12.8 to 6.8)
61–70	M	8.1 (6.0–10.1)	6.6 (5.0–8.3)	10.3 (8.2–12.3)	–9.2 (–12.3 to –6.0)
	F	8.4 (6.5–10.4)	7.2 (5.7–8.8)	11.0 (9.1–13.0)	–7.5 (–10.5 to –4.5)
71–80	M	9.1 (6.9–11.3)	7.8 (6.1–9.6)	11.7 (9.5–13.9)	–11.6 (–14.9 to –8.3)
	F	9.0 (7.0–11.0)	8.6 (7.1–10.2)	12.1 (10.2–14.1)	–9.1 (–12.2 to –6.0)
≥ 81	M	11.4 (8.5–14.3)	11.5 (9.3–13.7)	14.4 (11.6–17.1)	–12.5 (–16.7 to –8.3)
	F	15.2 (12.8–17.5)	14.7 (12.9–16.5)	17.5 (15.2–19.8)	–19.1 (–22.6 to –15.6)

PROM = patient-reported outcome measures, ANCOVA = analysis of covariance, SMFA = Short Musculoskeletal Function Assessment, EQ-VAS = EuroQol-Visual Analogue Scale, M = male, F = female, CI = 95% confidence interval.

a ANCOVA with fixed effect for age groups, sex, interaction between age groups and sex, mechanism of injury, open or closed injury, fracture classification, and day 0 value.

### PROMs and fracture classification

All patient groups (AO/OTA A1–C3) demonstrated a significant impairment in mean delta value (Table 4). The largest impact was seen in patients with type C fractures. C2 fractures had the largest impairment on all PROMs. The type A fractures had the smallest impact on PROMs and patients with A1 fractures had the least impairment of all fracture groups. Overall, a pattern with an increasingly negative impact on PROMs was seen with increasing severity of fracture type (Table 4). C fractures had a 1.2–1.9 points larger impairment of the SMFA indices and sub-category Mobility than B fractures, and B fractures had a 2.0–2.9 points larger impairment than A fractures.

**Table 4:** Mean and difference in mean delta values (∆) with (CI) of PROM with ANCOVA, 1 year after ankle fracture compared with day 0 in relation to the AO/OTA-classification

AO/OTA-classification	Bother (CI)	∆SMFA-index Dysfunction (CI)	Mobility (CI)	∆EQ-VAS (CI)
A1 ^a^	5.8 (3.8 to 7.7)	5.0 (3.4 to 6.6)	6.8 (4.9 to 8.8)	–8.4 (–11.4 to –5.4)
A2 ^a^	9.7 (7.5 to 11.9)	8.0 (6.3 to 9.8)	11.5 (9.4 to 13.7)	–10.2 (–13.6 to –6.9)
A3 ^a^	10.1 (7.1 to 13.1)	9.2 (6.8 to 11.5)	12.8 (9.9 to 15.8)	–9.9 (–14.5 to –5.3)
B1 ^a^	6.9 (5.0 to 8.8)	5.9 (4.4 to 7.4)	8.3 (6.4 to 10.2)	–7.9 (–10.8 to –5.0)
B2 ^a^	11.7 (9.7 to 13.6)	9.6 (8.0 to 11.1)	13.4 (11.5 to 15.3)	–10.8 (–13.8 to –7.8)
B3 ^a^	13.3 (11.4 to 15.3)	11.1 (9.5 to 12.6)	15.9 (14.0 to 17.8)	–13.5 (–16.5 to –10.5)
C1 ^a^	8.9 (6.8 to 11.1)	7.4 (5.7 to 9.1)	10.6 (8.6 to 12.7)	–9.6 (–12.8 to –6.4)
C2 ^a^	14.6 (12.3 to 16.9)	11.6 (9.7 to 13.4)	16.2 (13.9 to 18.5)	–14.1 (–17.6 to –10.6)
C3 ^a^	11.8 (9.5 to 14.2)	9.5 (7.7 to 11.4)	13.8 (11.5 to 16.1)	–11.3 (–14.8 to –7.8)
A1 vs. A2 ^a^	3.9 (2.4 to 5.4)	3.1 (1.9 to 4.2)	4.7 (3.3 to 6.2)	–1.8 (–3.9 to 0.3)
A2 vs. A3 ^a^	0.5 (–2.3 to 3.2)	1.1 (–1.0 to 3.2)	1.3 (–1.4 to 4.0)	0.3 (–3.8 to 4.5)
B1 vs. B2 ^a^	4.8 (4.0 to 5.7)	3.6 (2.9 to 4.3)	5.1 (4.2 to 5.9)	–2.9 (–4.2 to –1.6)
B2 vs. B3 ^a^	1.6 (0.6 to 2.7)	1.5 (0.7 to 2.3)	2.5 (1.5 to 3.5)	–2.7 (–4.2 to –1.2)
C1 vs. C2 ^a^	5.7 (3.7 to 7.6)	4.2 (2.6 to 5.7)	5.6 (3.6 to 7.5)	–4.5 (–7.3 to –1.7)
C2 vs. C3 ^a^	–2.8 (–4.9 to –0.7)	–2.0 (–3.7 to –0.4)	–2.4 (–4.5 to –0.3)	2.9 (–0.2 to 5.9)
A total ^b^	7.7 (5.8 to 9.7)	6.6 (5.1 to 8.1)	9.2 (7.3 to 11.1)	–9.4 (–12.4 to –6.5)
B total ^b^	10.2 (8.3 to 12.1)	8.5 (7.1 to 10.0)	12.1 (10.2 to 14.0)	–10.3 (–13.2 to –7.5)
C total ^b^	12.1 (10.1 to 14.1)	9.8 (8.2 to 11.3)	14.0 (12.0 to 15.9)	–11.8 (–14.8 to –8.8)
A vs. B ^b^	2.5 (1.8 to 3.2)	2.0 (1.4 to 2.5)	2.9 (2.2 to 3.6)	–0.9 (–1.9 to 0.1)
B vs. C ^b^	1.9 (1.0 to 2.8)	1.2 (0.5 to 1.9)	1.9 (1.0 to 2.7)	–1.5 (–2.7 to –0.2)

a ANCOVA with fixed effect for sex, age groups, mechanism of injury, open or closed injury, fracture classification (type and group) and day 0 value.

b ANCOVA with fixed effect for sex, age groups, mechanism of injury, open or closed injury, fracture classification (type) and day 0 value.

For Abbreviations, see Table 3.

The same tendency was seen in the fracture groups (1, 2, 3), at least for the PROM measuring function (i.e., SMFA), where group 3 for type A and type B fractures had a larger impairment than groups 2 and 1. The exception was C2 fractures, which had a larger impairment in PROMs than both C1 and C3 fractures.

### PROMs, energy level, and open fracture

A closed, low-energy caused fracture had the smallest impact on PROMs, rendering a 6.6–9.8 points impairment for the SMFA indices and sub-category Mobility, and 8.3 points impairment for EQ-VAS (Table 5). An open fracture caused by high-energy trauma gave the largest impairment on PROMs, and was significant on all PROM scores (SMFA indices and sub-category Mobility 16.7–23.0 and EQ-VAS 18.4). An open, high-energy caused fracture generated a 6.7–11.1 points larger impairment in the SMFA indices and sub-category Mobility, and 9.0 points larger impairment in EQ-VAS, compared with a closed, high-energy caused fracture. The same pattern was seen when comparing open and closed fractures in the group of low-energy caused fractures in the SMFA indices and sub-category Mobility.

**Table 5:** Mean and difference in mean delta values (∆) with (CI) of PROM with ANCOVA, 1 year after ankle fracture compared with day 0 in relation to type of trauma and open or closed injury

		∆SMFA-index		
Factor	Bother (CI)	Dysfunction (CI)	Mobility (CI)	∆EQ-VAS (CI)
High-energy^a^				
Open (n = 33)	17.2 (11.9–22.4)	16.7 (12.6–20.7)	23.0 (17.9–28.0)	–18.4 (–25.6 to –11.2)
Closed (n = 418)	10.5 (9.0–12.0)	8.3 (7.2–9.5)	11.9 (10.5–13.3)	–9.4 (–11.5 to –7.3)
Open vs. closed	6.7 (1.2–12.1)	8.4 (4.2–12.5)	11.1 (5.9–16.3)	–9.0 (–16.4 to –1.5)
Low-energy^a^				
Open (n = 175)	12.5 (10.2–14.8)	9.7 (7.9–11.5)	13.2 (11.0–15.5)	–11.3 (–14.6 to –8.0)
Closed (n = 10,062)	8.2 (7.7–8.7)	6.6 (6.2–7.0)	9.8 (9.3–10.2)	–8.3 (–9.0 to –7.6)
Open vs. closed	4.3 (2.0–6.6)	3.1 (1.3–4.9)	3.5 (1.3–5.7)	–3.0 (–6.3 to 0.3)
High-energy^b^	12.9 (11.2–14.5)	10.5 (9.2–11.8)	14.6 (13.0–16.2)	–11.6 (–9.4 to –8.5)
Low-energy^b^	10.4 (9.3–11.5)	8.5 (7.6–9.3)	12.0 (10.9–13.0)	–10.1 (–11.7 to –8.5)
High- vs. low-energy^b^	2.5 (1.1–3.9)	2.1 (0.9–3.2)	2.6 (1.2–4.1)	–1.5 (–3.6 to 0.5)

a ANCOVA with fixed effect for mechanism of injury, open or closed injury, interaction between mechanism of injury and open or closed injury, sex, age groups, fracture classification, and day 0 value.

b ANCOVA with fixed effect for sex, age groups, mechanism of injury, open or closed injury, fracture classification, and day 0 value.

For Abbreviations, see Table 3.

## Discussion

We aimed to analyze PROMs in patients with ankle fractures in relation to the AO/OTA fracture classification and showed that PROMs after ankle fractures are associated with fracture morphology. Patients with C fractures reported the poorest outcome, while A fractures had the least negative effect when outcome was adjusted for covariates and assessed with PROMs. Trimalleolar fractures had a poorer outcome than bimalleolar fractures, which in turn had a poorer outcome than unimalleolar fractures.

Until now, studies reporting on PROMs where all types of ankle fracture are represented have been lacking. Previous small studies have reported an impairment in PROMs in patients following ankle fractures, but they have also indicated that the fracture pattern did not affect outcome [6,7,16,17]. However, those studies focused solely on either surgically or non-surgically treated fractures or were limited by including only 1 fracture type or group. A single-center study by Chong et al. reported data on 180 surgically treated patients with unstable ankle fractures, where approximately half the patients had filled in questionnaires and they reported a deterioration in the PROM scores—the Olerud–Molander Ankle Score and the Lower Extremity Functional Scale—at 2 and 5 years post-fracture [6]. The number of fractured malleoli had a correlation to remaining potential complications such as stiffness, swelling, and pain but did not affect PROMs. Audet et al. reported that female sex, younger patients, and patients with a high BMI displayed a deterioration in PROMs when the SMFA was used to evaluate outcome in 416 surgically treated ankle fractures with a mean follow-up period of 6 years [16]. The use of tobacco or alcohol was also shown to affect outcome negatively. However, fracture pattern and open fractures were not shown to affect PROMs in their study.

### PROMs in relation to age and sex

The largest impairment in PROMs was seen among the oldest patients (aged ≥ 81). For patients younger than 81 years, there was a relatively equal change in PROMs among the different age groups and for both sexes. An exception was the Bother index, where the youngest patients (18–30 years of age), among both men and women, had a larger impairment than other age groups aged < 81.

Many of the older patients are likely to have physical limitations before their injury and might therefore be even more negatively affected by an ankle fracture, with the need for a longer rehabilitation period than a younger patient. Hence the greater impairment found in PROMs 1 year after injury for the oldest patients in this study. This general frailty in many of the oldest patients with ankle fractures likely renders a need for contributions from society such as domestic service or retirement homes following their fracture.

One might assume that increasing age leads to higher impairment in PROMs and that the youngest patients would have the least impairment in PROMs. However, younger patients had impairment in PROMs comparable to all other age groups except the very oldest (aged ≥ 81). Although most young patients have no physical limitations before their injury, they also have high functional demands and high expectations of their injured limb. This may correspond to the relatively high reported impairment in PROMs in these groups. Another factor negatively affecting PROMs is the higher incidence of high-energy trauma and open fractures seen in younger patients [18]. Our study demonstrates that both open injuries and high-energy trauma had a statistically significant negative effect on outcome, and even more so when combined. This combination is likely to include more severe soft-tissue damage, which might affect outcome negatively.

### PROMs and fracture classification

A significant increasing negative impact on PROMs was seen with increasing fracture severity according to the AO/OTA classification system. The number of fractured malleoli reflects the severity of the injury to both the skeleton and the ligaments around the ankle, which seems to affect the outcome. The other prognostic factor demonstrated in this study was the height of the fibular lesion, with a higher fracture location on the fibula predisposing to a poorer functional outcome measured by SMFA. Only 1 exception was noted to this prognostic ability of the AO/OTA alphanumeric classification system. The C2 fracture had a poorer outcome than the C3 fracture on all PROMs. A high fibular fracture is associated with a potential higher risk of more severe syndesmotic injuries and associated membrane injuries than suprasyndesmotic fractures. This would lead to a higher degree of instability and thereby a more severe injury. However, Rydberg et al. demonstrated that patients with C2 fractures had the lowest mean age among all ankle fracture types and C2 was also the fracture type with the largest proportion of open fractures [18]. This could be one explanation as to why C2 fractures in this study had a greater impairment in PROMs than C3 fractures, because open fractures had a major negative effect on PROMs. Another possible explanation is that stable high fibular fractures due to a direct injury mechanism could mistakenly have been registered as C3 fractures.

The finding of a deteriorating PROM with increasing fracture severity might support the hypothesis that a PROM is an adequate method for studying outcome after ankle fractures. SMFA is validated and with high reliability when used in patients with musculoskeletal disorders and injuries. In a previous study on ankle fractures with a 6-week follow-up, McCreary et al. concluded a minimum clinically important difference (MCID) of 7 to determine a clinically significant result [19]. Reasonably, a smaller impairment might also be relevant over time, as in our study with a 1-year follow-up time.

### Strength and limitations

*Strengths.* This is a population-based cohort, with prospectively collected data. Although there was a low response rate, comparison between the responders and the non-responders showed only a slightly higher proportion of women and a marginally higher mean age in the responder cohort and the distribution of fractures was similar. These findings correspond with the findings in a study by Juto et al., which concluded that age and sex were factors that affected the response rate to PROMs and that the highest responsiveness was seen among women between 60 and 69 years of age [20].

*Limitations.* The recall technique that was used when patients reported their pre-injury functional level probably increases with increasing time between fracture and the reporting of the pre-injury function. Even though patients were only sent PROM questionnaires up to 4 weeks after their fracture occurred, it is not known when they actually completed the questionnaires. A late response can lead to a risk for recall bias where it is known that patients tend to forget their actual functional level before the fracture, and therefore estimate higher function. Finally, we were not able to adjust for several residual confounders, such as high BMI or tobacco and alcohol use, as they are not registered in the SFR [16].

### Conclusions

The AO/OTA classification for ankle fractures correlates with outcome, as more complex fractures were associated with poorer patient-reported outcome. Other factors that potentially negatively influencing the outcome were high-energy caused and open fractures.

## Notes

CS: designed the study, performed the data acquisition and the statistics, and wrote the manuscript. MM, DW, OW, CB: designed the study, revised the manuscript. JE: revised the manuscript and supervised the statistical analyses.

The authors would like to thank all the orthopedic surgeons for registering fractures in the SFR, making this study possible.

Handling co-editors: Jeppe Vejlgaard Rasmussen and Philippe Wagner

*Acta* thanks Lars Gunnar Johnsen and Aleksi Reito for help with peer review of this manuscript.
